# Astragaloside IV Inhibits Triglyceride Accumulation in Insulin-Resistant HepG2 Cells via AMPK-Induced SREBP-1c Phosphorylation

**DOI:** 10.3389/fphar.2018.00345

**Published:** 2018-04-16

**Authors:** Chunyi Wang, Yan Li, Mengjiao Hao, Weimin Li

**Affiliations:** ^1^School of Pharmaceutical Sciences, Guangzhou University of Chinese Medicine, Guangzhou, China; ^2^Integrated Chinese and Western Medicine Postdoctoral Research Station, Jinan University, Guangzhou, China; ^3^Shenzhen Institute of Geriatrics, Shenzhen, China

**Keywords:** astragaloside IV, triglyceride, insulin resistance, AMPK, SREBP-1c, phosphorylation of SREBP-1c at Ser372

## Abstract

**Objective:** Insulin resistance (IR) is a risk factor for non-alcoholic fatty liver disease (NAFLD), which is characterized by lipid accumulation in hepatocytes. AMP-activated protein kinase (AMPK)-induced sterol regulatory element binding protein-1c (SREBP-1c) phosphorylation is crucial for proper regulation of lipid metabolism in the liver. Astragaloside IV (AST-IV) was found to decrease lipid accumulation in hepatocytes by activating AMPK, which is required to regulate lipid metabolism in liver tissue by inducing SREBP-1c phosphorylation.

**Method:** To evaluate the direct effect of AST on lipid accumulation in hepatocytes with IR and elucidate the underlying mechanisms, we induced IR in HepG2 cells, and used compound C and 5-aminoimidazole-4-carboxamide-1-β-D-ribofuranoside (AICAR) (an AMPK inhibitor and agonist, respectively) as control substances. We evaluated glucose, triglyceride (TG), and non-esterified fatty acid (NEFA) production, as well as *SREBP-1c* transcription, SREBP-1c protein expression, and downstream gene expression with or without the presence of AST. We also investigated whether phosphorylation of SREBP-1c at Ser372 was required for AST function.

**Results:** We found that AST attenuated IR and lipid accumulation in HepG2 cells. As an AMPK activator, AST promoted gene expression and activation of AMPK by increasing phosphorylation of AMPKa. AST also inhibited translocation of SREBP-1c into the nucleus of insulin-resistant HepG2 cells by inducing phosphorylation of SREBP-1c at Ser372.

**Conclusion:** This study demonstrated that AST attenuates IR and lipid accumulation in HepG2 cells by regulating AMPK-dependent phosphorylation of SREBP-1c at Ser372, suggesting AST as a promising drug for treating hepatic steatosis.

## Introduction

Non-alcoholic fatty liver disease, which includes NAFL, and NASH, is characterized by lipid accumulation in the hepatocytes of individuals who do not consume excessive amounts of alcohol. NAFLD can progress to fibrosis, cirrhosis, and even hepatocellular carcinoma (Bellentani et al., 2000; Fan et al., 2007). The prevalence of NAFLD has gradually increased. Previously studies reported that 27% of urban Chinese adults had NAFLD (Fan, 2013; Lankarani et al., 2013), and that prevalence increased to 58–74% in an obese population (Luyckx et al., 1998). Although the underlying pathological mechanism of NAFLD remains unclear, a causal relationship between lipid accumulation in hepatocytes and IR has been reported during the development of NAFLD, obesity, type 2 diabetes, and dyslipidemia (Petersen et al., 2006; Loomba et al., 2012). Lipotoxicity induced by high levels of free fatty acids (FFA) and cholesterol metabolites in the liver results in increased levels of oxidative stress, endoplasmic reticulum stress, and mitochondrial dysfunction, all of which impair glucose metabolism and glycogen synthase activity (Kumashiro et al., 2011; Buzzetti et al., 2016). High levels of FFA and cholesterol metabolites can also activate the PKC and c-JNK-1 pathways, and lead to dysfunction of insulin receptor substrate 1 (IRS)-1 and IRS-2 tyrosine (Samuel et al., 2004, 2007; Kumashiro et al., 2011). In Asian populations, IR is an independent risk factor for development of NAFLD among individuals without metabolic syndrome (Fan et al., 2007; Fan, 2013). Because no effective and safe treatment is available for NAFLD, except for lifestyle intervention-mediated weight loss, it is extremely important to develop new and effective therapeutic methods.

Astragaloside IV [AST-IV; also known as Astragalosides A (Yu, 1986)] is extracted from *Astragalus membranaceus*, and the major bioactive ingredient of the *Astragalus* species (Ren et al., 2013; Li et al., 2017). AST has multiple pharmacological properties, including immunomodulatory effects (Wang et al., 2002; Ren et al., 2013; Li et al., 2017), as well as anti-inflammatory (Lee et al., 2013; Zhang and Frei, 2015) and antiviral (Chen et al., 2012) effects. Animal studies have reported that AST helps to regulate lipid and carbohydrate metabolism. AST was shown to reduce free fatty acid (FFA)-induced lipid accumulation in rat hepatocytes (Jiang et al., 2008), and in a type 2 diabetic rat model, a *Astragali Radix* extract lowered IRce and helped ameliorate fatty livers (Jiang et al., 2008). A recent study reported that AST attenuates FFA-induced ER stress and lipid accumulation in hepatocytes via AMPK activation (Zhou et al., 2017). Astragaloside IV was reported to improve lipid metabolism in obese mice by alleviating leptin resistance and regulating the mouse thermogenic network (Wu et al., 2016). Those study results suggest that AMPK activation in hepatocytes may be an underlying mechanism for the effect of AST on lipid metabolism.

AMP-activated protein kinase is a member of the serine/threonine kinase family, which plays a crucial role in regulating energy metabolism. AMPK becomes activated by phosphorylation of Thr172 located in its α subunit. Activated AMPK can inhibit fatty acid synthesis (Su et al., 2012; Zhou et al., 2017) by targeting SREBP-1c, which is a pivotal transcription factor involved in the transcription of lipogenic genes, including *ACC1*, as well as genes encoding for *FAS* (Serviddio et al., 2013; Nakamura et al., 2014) and *SCD1* (Su et al., 2012; Zhou et al., 2017).

Sterol regulatory element binding protein-1c is characterized as conserved substrates of AMPK. AMPK regulates SREBP-1c proteolytic nuclear translocation by phosphorylating SREBP-1c at Ser372 (Li et al., 2011). In HepG2 cells cultured under conditions of a high glucose or high insulin concentrations, AMPK represses SREBP-1c-induced downstream gene expression. This repression was shown to reduce lipogenesis and lipid accumulation in HepG2 cells (Li et al., 2011). The AMPKα subunit strongly associates with and highly phosphorylates the precursor and nuclear forms of SREBP-1c (Li et al., 2011). Taken together, these findings indicate that AMPK activation serves to attenuate hepatic lipid accumulation, and suggest AMPK as a potential therapeutic target for treating hepatic steatosis.

Zhou et al. (2017) demonstrated that AST attenuates FFA-induced ER stress and lipid accumulation in hepatocytes in manners dependent on the level of AMPK. While the effects of AST have also been studied in other cell types (Xu et al., 2006; Jiang et al., 2008; Zhao et al., 2015), few studies have focused on the effect of AST on regulating IR in hepatocytes. In this study, we hypothesized that AST inhibits lipid accumulation in insulin-resistant hepatocytes by activating AMPK, and thereby inducing phosphorylation of SREBP-1c. We then performed a series of experiments designed to test our hypothesis.

## Materials and Methods

### Establishment of Insulin Resistance in HepG2 Cells

HepG2 cells were cultured in low sugar DMEM (Invitrogen, Carlsbad, CA, United States) supplemented with 10% fetal bovine serum in an incubator (37°C and 5% CO_2_) (Thermo Fisher Scientific, Waltham, MA, United States). At 2 days after confluence, 30 mM glucose (Mackin, Shanghai, China) and 100 nM insulin (Mackin, Shanghai, China) were added to the growth medium, and the cells were incubated for 24 h to induce IR. After induction, the HepG2 cells were divided into different treatment groups, which included a control group, insulin-resistant (IR) group, insulin-resistant + AST (IR+AST) group, insulin-resistant + 5-aminoimidazole-4-carboxamide-1-β-D-ribofuranoside (IR+AICAR) group, insulin-resistant + compound C (IR+C) group, insulin-resistant + AST + compound C (IR+AST+C) group, and an insulin-resistant + astragaloside IV + AICAR (IR+AST+AICAR) group. AST (MCE, Princeton, NJ, United States, Cat. No. HY-N0099) was diluted with DMEM to a final concentration of 50 μg/mL prior to use, and compound C (also referred to a dorsomorphin, Selleck, Houston, TX, United States, Cat. No. S7840) was diluted with DMEM to a final concentration of 10 μM. ACAIR (refer to 5-aminoimidazole-4-carbox-amide-1-β-D-ribofuranoside, MCE, Princeton, NJ, United States, Cat. No. HY-13417) was diluted with DMEM medium to a final concentration of 2 mM.

### Lentiviral Transfection

Lentivirus packaging SREBP-1c S372A wild-type plasmids and SREBP-1c S372A mutant plasmids were synthesized by Shanghai Genepharma Co., Ltd. One day prior to transfection, 1×10^5^ HepG2 cells were placed into each well of a 6-well plate containing 1 mL of complete medium per well, and cultured overnight in a 37°C incubator containing 5% CO_2_. On the second day, 100 μL of lentivirus stock solution was diluted with 900 μL of complete medium; after which, Polybrene was added to a final concentration of 5 μg/mL. Next, the cell culture medium was replaced by the lentivirus dilution, and cells were cultured at 37°C for 24 h in a 5% CO_2_ atmosphere. On the third day, the lentivirus dilution was removed and 100 μL of fresh complete medium was added to each well of the 6-well plate. The cells were then incubated for 48 h at 37°C in 5% CO_2_ atmosphere to evaluate cell transfection efficiency. The transfected cells were harvested for use in subsequent experiments.

### Western Blot Assay

Treated HepG2 cells were washed two times with phosphate buffered saline (PBS) twice prior to collection. The collected cells were then lysed in RIPA buffer, and centrifuged for 30 min at 13,000g and 4°C. The supernatant fraction was collected and heated with a fourfold volume of loading buffer at 95°C for 5 min. Electrophoresis was performed on a 12% SDS–polyacrylamide gel. After electrophoresis, the separated protein bands were transferred onto 0.45 μm PMSF membranes for 1 h, and the membranes were then blocked with 5% skim milk for 1 h. The PMSF membranes were then exposed to primary antibodies (anti-AMPKα, Cell Signaling Technology, Danvers, MA, United States; anti-P-Thr172 AMPKα, Cell Signaling Technology, United States; anti–SREBP-1c, Abcam, Cambridge, MA, United States; anti-P-Ser372-SREBP-1c, Cell Signaling Technology, lot. 9874, anti-FAS, Abcam, United States; anti-P-Ser731-IRS-2, Abcam, United States; anti-IRS-2, Cell Signaling Technology, United States; anti-ACC1, Cell Signaling Technology, United States; anti-p-ser79 ACC1, Cell Signaling Technology, United States; anti-SCD1, Abcam, United States; anti-GAPDH, Shanghai Kangcheng) in blocking buffer at 1:500 or 1:1000 dilutions overnight at 4°C. The membranes were then incubated with a secondary horseradish-conjugated antibody (Boster, Shanghai, China) at a 1:5000 dilution for 1 h. The immunostained proteins were visualized by enhanced chemoluminescence (ECL), and scanned using a bio-imaging analyzer (Bio-Rad, Hercules, CA, United States). A Beyotime nucleoprotein extraction kit (Beyotime, Beijing, China, Cat. No. P0027) was used to extract nuclear proteins for detection of SEREBP-1c.

### Glucose Consumption Assay

Glucose consumption assays were performed using a D-Glucose Assay kit (Rongsheng Biotech, Shanghai, China) according to the manufacturer’s instructions. The growth medium from HepG2 cells in the different treatment groups was spun down in a centrifuge column, and the glucose concentrations before and after the 24-h treatment were evaluated using the kits.

### Oil Red O Staining

Cells were washed with ice-cold PBS and fixed with 10% formaldehyde solution for 15 min at 25°C. The cells were then washed with PBS and incubated with reagents in an Oil Red O Staining kit (Yope Biotechnology, Shanghai, China) for 30 min at 25°C. Next, the cells were briefly washed with 75% ethanol to remove any unbound dye and observed under a Leica DMI4000B inverted microscope after being washed with PBS.

### Immunofluorescence Assay

Cells were fixed and processed into paraffin-embedded slides. After antigen retrieval, 3% H_2_O_2_ was used to inactivate endogenous peroxidase, and the slides were then blocked with 1% BSA/PBS solution. For immunofluorescence assays, the slides were incubated with primary antibodies over night at 4°C; after which, they were incubated with a biotinylated–modified secondary antibody at for 1 h at 25°C. Next, conjugated HRP-labeled streptavidin (Dako, Glostrup, Denmark) was added, and the slides were incubated for 30 min at 25°C. Diaminobenzidine (DAB; Sigma, St. Louis, MO, United States) was used as the chromogen. Photographs of the stained slides were taken with an FV10i confocal microscope (OLYMPUS, Japan).

### Real-Time RT-PCR

Total RNA was extracted from treated cells with Trizol Reagent (TaKaRa Biotechnology, China). cDNA was synthesized from 1 μg of total RNA with a reverse transcriptase kit (DBI Bioscience, Newark, DE, United States). Primers used in the real-time RT-PCR (rt-PCR) assay are listed in **Table 1**. GAPDH served as an internal reference. SYBR Green qPCR Master Mix (DBI Bioscience, United States) was used for rt-PCR amplification. The cycling conditions were denaturation at 95°C for 2 min followed by 40 repeated annealings at 94°C for 20 s, and extension at 58°C for 20 s. Due to exponential amplification of the target gene as well as a calibrator, fold-changes in gene expression were calculated using the 2^-ΔΔ*C*_t_^ method.

**Table 1:** List of primers used in the study.

ID	Sequence (5′–3′)
GAPDH F	TGTTCGTCATGGGTGTGAAC
GAPDH R	ATGGCATGGACTGTGGTCAT
AMPKa1 F	TTGAAACCTGAAAATGTCCTGCT
AMPKa1 R	GGTGAGCCACAACTTGTTCTT
AMPKa2 F	CTGTAAGCATGGACGGGTTGA
AMPKa2 R	AAATCGGCTATCTTGGCATTCA
SREBP-1c F	CGGAACCATCTTGGCAACAGT
SREBP-1c R	CGCTTCTCAATGGCGTTGT
FAS F	TCTGGTTCTTACGTCTGTTGC
FAS R	CTGTGCAGTCCCTAGCTTTCC
ACC1 F	TCACACCTGAAGACCTTAAAGCC
ACC1 R	AGCCCACACTGCTTGTACTG
SCD1 F	TTCCTACCTGCAAGTTCTACACC
SCD1 R	CCGAGCTTTGTAAGAGCGGT


**SREBP-1c, sterol regulatory element binding protein-1c; AMPK, AMP-activated protein kinase; SCD1, stearoyl-coenzyme A desaturase 1; FAS, fatty acid synthase*.*

### ELISA Assay

Levels of TGs and NEFAs were measured using antigen-based sandwich ELISA kits for TGs and NEFAs, respectively (Elabscience, Wuhan, China). Absorbance at 450 nm was measured using a microplate spectrophotometer (Thermo Scientific Multiskan GO).

### Recombinant Plasmid Construction

The 5′UTR sequence of SREBP-1c (NCBI Gene ID: 6720) was retrieved from the GenBank Database. PCR was performed to amplify a fragment encoding the SREBP-1c sequence from template DNA by using the forward primer 5′-GGGGTACCCCAGCCTGGCCAAAATG-3′ and reverse primer 5′-CCGCTCGAGAGCTCTGCTGGGCGGTC-3′. The PCR product was then cloned into the Kpnl/Xhol sites of the pGL3 vector (Promega). The pGL3-IRS2 was prepared using the same method, but with the forward primer being 5′-GGGGTACCCACCTACAGGGCAAAGAACTAAA-3′and reverse primer being 5′-CCGCTCGAGGAGGTGAGGGCTTCCTAGAGTT-3′. Mutations of the Ser 372 binding sites in the SREBP-1c sequence were created using a KOD-Plus-Mutagenesis Kit (Agilent Technologies, Santa Clara, CA, United States) according to the manufacturer’s protocol. All plasmids were confirmed by restriction enzyme digestion and sequencing.

### Dual-Luciferase Reporter Assay

The pGL3-SREBP-1c and pGL3-IRS2 were transiently transfected into HepG2 cells. After 48 h, the cells were harvested and lysed. Cellular luciferase activity was measured using a Dual-Luciferase^®^ Reporter (DLR^TM^) Assay System (Promega, Madison, WI, United States). One day prior to transfection, the cells were inoculated into 24-well plates at a density of 2 × 10^4^ cells/well with medium containing 10% FBS. Transfection was conducted when cell confluence reached 50∼60%. The cells were washed twice with PBS and 300 μL of serum-free basal medium was added to each well. The plate was then placed in a 5% CO_2_, 37°C incubator. In each pore, 1 μL of Lipofectamine 2000 was diluted with a serum-free base medium to a final volume of 50 μL; after which, 4 μg of the different plasmids was added to each well. DMEM-high glucose medium was then added to a total volume of 50 μL. Next, 100 μL of each transfection mixture was shaken in a 24-well plate, which was then incubated in a 5% CO_2_, 37°C incubator for 5 h. The transfection medium was then replaced with fresh complete medium. After transfection for 48 h, the culture medium was replaced and the cells were washed twice with PBS. A 100 μL volume of Passive Buffer Solution (PLB) was added to each well, and the plate was shaken at room temperature for 15 min to collect cell lysate. Next, 20 μL of cell lysate was added to a luminescent plate, and the background value (2s) was read using a GloMax bioluminescence detector. LAR II working solution (100 μL) was added to each sample and rapidly mixed for 2 s. After reading the value, 100 μL of Stop & Glo^®^ Reagent was added to each sample, and the sample was placed in the luminous detector for 2 s.

### CCK8 Assay

The CCK8 assay was optimized for the cell lines used in our experiments. Briefly, HepG2 cells were treated with trypsin and then seeded into 96-well plates at a density of 3 × 10^3^ cells/well. After 24 h, AST was added to each well at final concentrations of 0.25, 0.5, 1.0, 10, 20, 40, and 80 μg/mL, respectively, and incubated with the cells. After 24, 48, and 72 h of AST treatment, 10 μL of CCK-8 (Sigma, United States) solution (5 g/L, dissolved in PBS) was added to each well and the plates were incubated for an additional 4 h. Cell proliferation was evaluated after 24, 48, and 72 h of AST treatment and recorded as the OD_450_ value of each well as measured with a microculture plate reader (BioTek, Winooski, VT, United States).

### Statistical Analyses

All results are expressed as the mean ± SD of data obtained from at least three independent experiments. All statistical analyses were performed using IBM SPSS Statistics for Windows, Version 19.0 (IBM Corp., Armonk, NY, United States). Student’s *t*-test was used to analyze differences between two groups, and one-way ANOVA was used to determine the significance of differences among multiple groups. *P*-values < 0.05 were considered statistically significant.

## Results

### AST Reduced Triglyceride Accumulation and Increased Glucose Consumption in Insulin-Resistant HepG2 Cells

**FIGURE 1:**
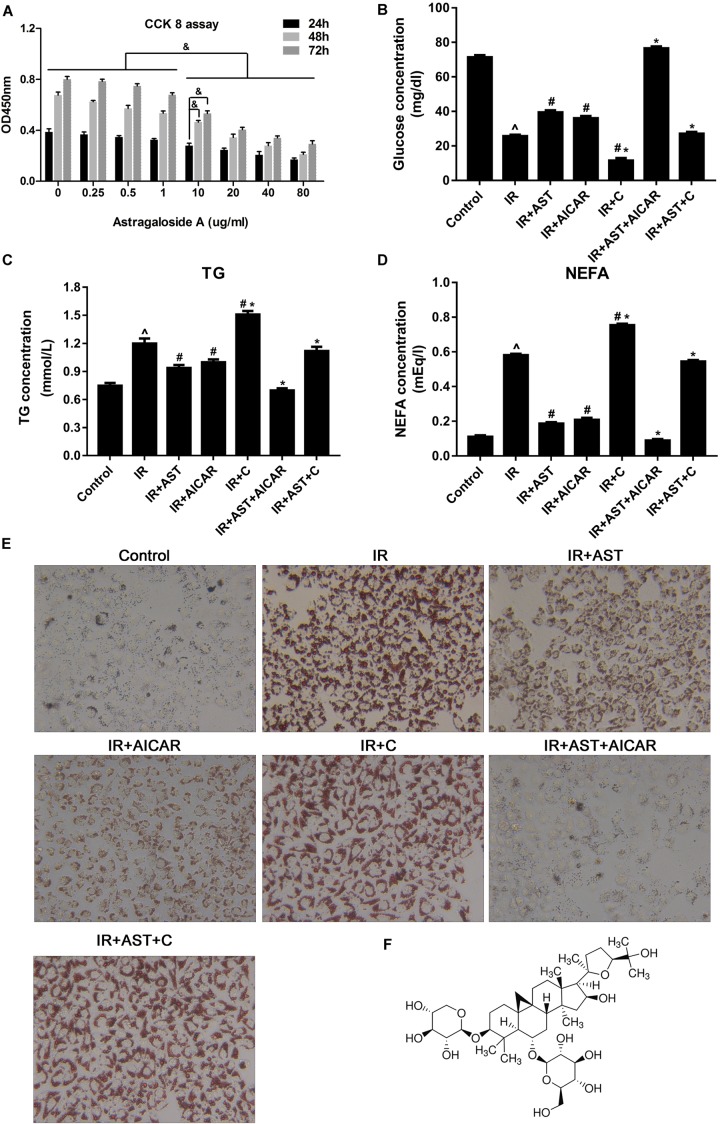
AMP-activated protein kinase (AMPK) activation by AST blunted dysfunction of lipid metabolism and insulin resistance in HepG2 cells. **(A)** Evaluation of AST cytotoxicity when incubated with cells at concentrations of 0.25, 0.5, 1.0, 10, 20, 40, and 80 μg/mL for 24, 48, and 72 h. AST improved glucose consumption **(B)** and decreased TG **(C),** and NEFA **(D)** levels in insulin-resistant HepG2 cells. **(E)** Representative gross morphology of a triglyceride accumulation in insulin-resistant HepG2 cells as visualized by Oil Red O staining; magnification: ×200^∗^. **(F)** Chemical structure of AST. AST, astragalosidea; C, compound C; TG, triglyceride; NEFA, non-esterified fatty acid; IR, insulin resistance. Date represent the mean SD, as determined by one-way ANOVA, ˆ*p* < 0.05 vs. the control group.^∗^*p* < 0.05 vs. the IR+AST group; #*p* < 0.05, vs. the IR group; ^&^*p* < 0.05.

To investigate the effect of AST on regulating lipid metabolism in insulin-resistant HepG2 cells, we performed CCK8 assays to evaluate the toxicity of different concentrations of AST to HepG2 cells for the purpose of identifying a suitable concentration for use in subsequent experiments. We found that a 10 μg/mL AST concentration produced no significant toxic effects in HepG2 cells (**Figure 1A**). We then induced IR in HepG2 cells by exposing them to high concentrations of glucose (30 mM) and insulin (100 nM) for 24 h.

As shown in **Figure 1B**, insulin-resistant HepG2 cells displayed significantly decreased levels of glucose consumption, while application of AST or AICAR (AMPK activators) blunted the reduction in glucose consumption. Additionally, HepG2 cells treated with a combination of AST and AICAR displayed a normal level of glucose consumption when compared with control cells, indicating that the combination of AST and AICAR had restored insulin sensitivity in the HepG2 cells (**Figure 1B**, *p* < 0.05). Cells treated with compound C (an AMPK inhibitor) displayed a significantly decreased level of glucose consumption when compared with consumption in all the other groups. These results indicated that AST might reverse IR in HepG2 cells via activation of AMPK (**Figure 1B**, *p* < 0.05).

Our analysis of TG and FFA production produced results consistent with of our analysis of glucose consumption, as application of either AST or AICAR reduced TG (**Figure 1C**, *p* < 0.05) and FFA (**Figure 1D**, *p* < 0.05) levels, a combination of AST and AICAR exerted a synergistic effect on TG (**Figure 1C**, *p* < 0.05) and FFA levels (**Figure 1D**, *p* < 0.05), and application of compound C blunted the effect of AST (**Figures 1C,D**). Oil Red O staining confirmed these findings, as decreased numbers of lipid droplets were found in HepG2 cells treated with either AST or AICAR alone or with AST plus AICAR (**Figure 1E**), and treatment with compound C decreased the effect of AST on lipid droplets. When taken together, these findings suggest that AST (**Figure 1F**) can attenuate lipid accumulation in insulin-resistant HepG2 cells.

### AMPK Activation by AST Inhibited the Accumulation of Nuclear SREBP-1c and Suppressed Expression of Relevant Target Genes

**FIGURE 2:**
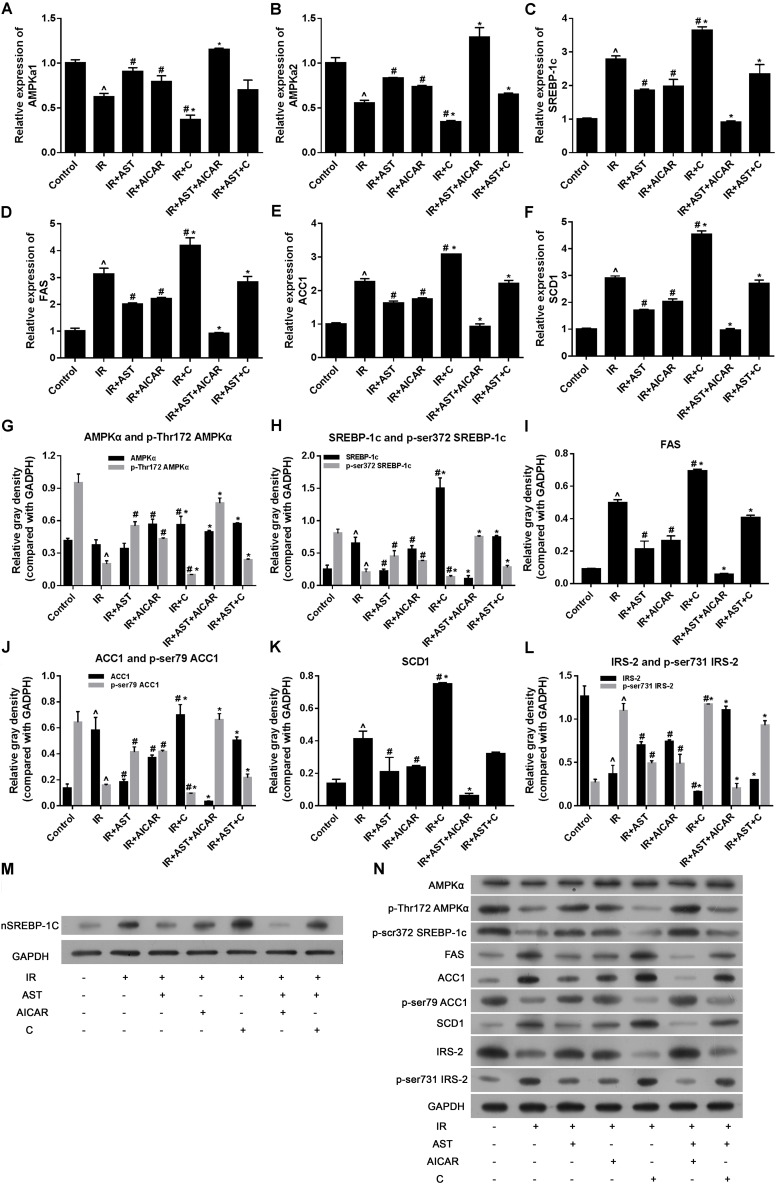
AMP-activated protein kinase activation by AST inhibited the expression of sterol regulatory element binding protein-1c (SREBP-1c) and SREBP-1c related genes. AST promoted AMPKα1 mRNA expression **(A)**, AMPKα2 mRNA expression **(B)**, and suppressed the expression of SREBP-1c **(C)**, and its downstream genes *FAS* **(D)**, *ACC1* **(E)**, and also AMPK downstream gene *SCD1* **(F)**. AMPK activation by AST promoted phosphorylation of AMPKα **(G)**, SREBP-1c **(H)**, ACC1 **(J)**, and IRS-2 **(L)**, and decreased the levels of FAS **(I)**, and SCD1 protein **(K)**. **(M)** Representative bands of SREBP-1c nuclear protein. **(N)** Representative bands of cytoplasmic proteins visualized in the immunoblotting assay. AST, astragaloside IV; C, compound C; IR, insulin resistance. Data represent the mean ± SD as determined by one-way ANOVA, ˆ*p* < 0.05 vs. the control group. ^∗^*p* < 0.05 vs. the IR+AST group; #*p* < 0.05 vs. the IR group.

AMP-activated protein kinase exists in its most active form when the α subunit is phosphorylated at Thr172 (P-Thr172 AMPKα) (Li et al., 2011). Therefore, we investigated the levels of mRNA for AMPKα1 and AMPKα2, as well as the levels of AMPKα and p-Thr172 AMPKα proteins after application of AST. The results revealed that application of AST or AICAR alone or in combination significantly increased AMPKα1 and AMPKα2 mRNA expression, while cells treated with compound C alone had significantly reduced levels of AMPKα1 and AMPKα2 mRNA when compared with other groups (*p* < 0.05). Furthermore, application of compound C eliminated the effect of AST on AMPKα1 and AMPKα2 mRNA expression (**Figures 2A,B**). Western blotting assays revealed that the levels of AMPKα and p-Thr172 AMPKα protein could be elevated by application of either AST or AICAR (**Figures 2G,N**).

As SREBP-1c is a transcription factor that plays a pivotal role in regulating lipid metabolism, we performed PCR and western blotting assays to detect the effect of AST-induced AMPK activation on SREBP-1c gene and protein expression, as well as its effect on downstream proteins including ACC1, FAS, and SCD1. PCR assay results revealed that either AST or AICAR alone could blunt the increases in mRNA levels for SREBP-1c, FAS, ACC1, and SCD1 in insulin-resistant HepG2 cells, while application of compound C alone further aggravated IR-induced changes in the expression of genes coding for SREBP-1c, FAS, ACC1, and SCD1, and also eliminated the effects of AST and AICAR (**Figures 2C–F**). Moreover, our western blotting assays confirmed the qPCR assay results (**Figures 2H–N**). These findings indicated that AST inhibits the activation of nuclear SREBP-1c and expression of its target genes.

**FIGURE 3:**
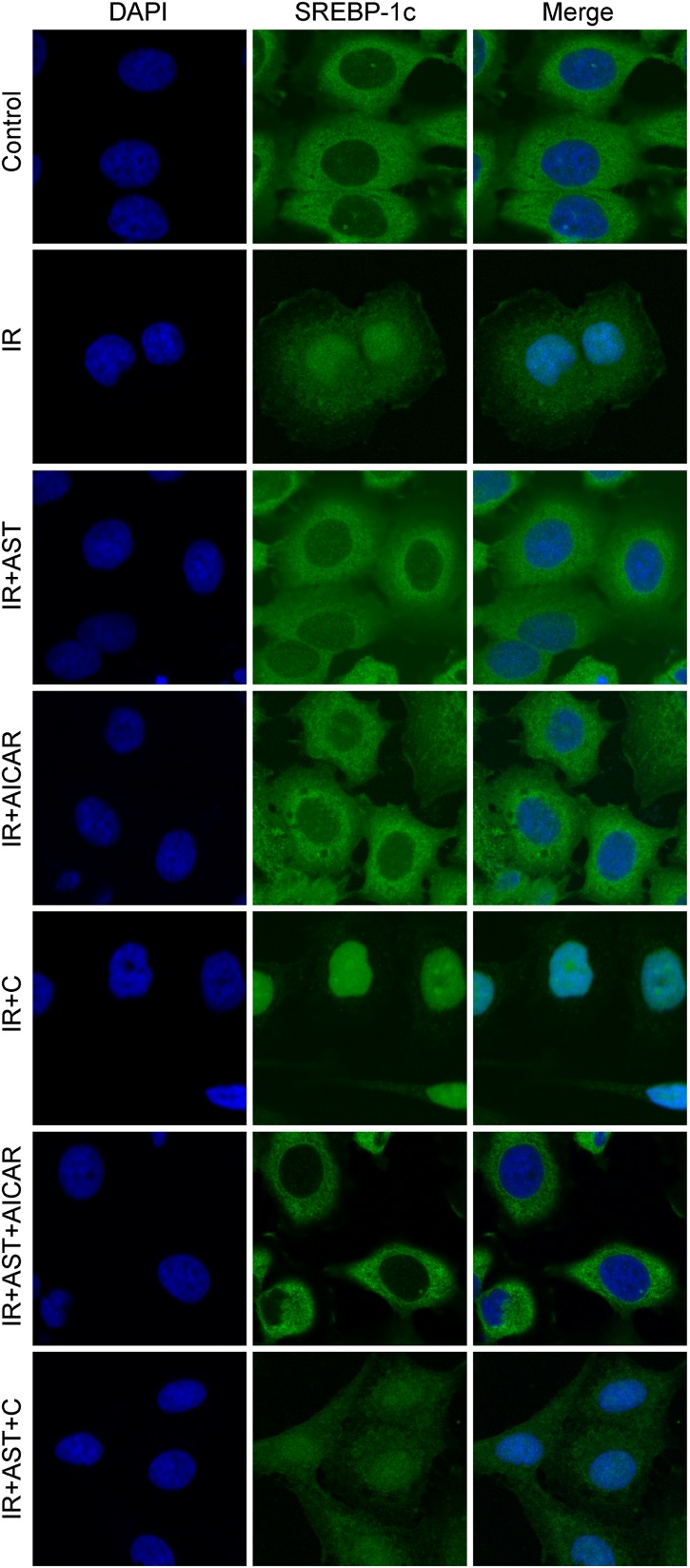
Astragaloside IV inhibited translocation of SREBP-1c into the nucleus. Insulin-resistant HepG2 cells treated with AST displayed a lower SREBP-1c fluorescence intensity in the nucleus. AST, astragaloside IV; C, compound C; IR, insulin resistance. Magnification: ×120.

Phosphorylation of SREBP-1c at Ser372 by activated AMPK was reported to suppress SREBP-1c nuclear translocation and repress expression of its target gene in hepatocytes exposed to high glucose concentrations, resulting in reduced levels of lipogenesis and lipid accumulation (Li et al., 2011). We evaluated the levels of nuclear SREBP-1c (nSREBP-1c) and P-Ser372 SREBP-1c, and found that activation of AMPK by application of AST or AICAR alone or in combination could blunt any increase of total SREBP-1c protein in the nucleus of insulin-resistant HepG2 cells, and inhibition of AMPK by compound C attenuated the effect of AST (**Figures 2G,M,N**). We also investigated the levels of ACC1, IRS-2, P-Ser79 ACC1, and P-Ser731 IRS-2 proteins, which are downstream target proteins of SREBP-1c. Our results showed that the effect produced by AST or AICAR alone might require the activation of P-Ser79 ACC1 (**Figure 2J**) and inhibition of P-Ser731 IRS-2 (**Figure 2L**) reactions, which resulted in reduced levels of ACC1 (**Figure 2J**) and increased levels of IRS-2 (**Figure 2L**). Immunofluorescence assays of SREBP-1c confirmed these findings, as AST or AICAR alone or in combination decreased SREBP-1c fluorescence intensity in the nucleus of IR HepG2 cells (**Figure 3**). These results indicate that activation of AMPK by AST suppresses the nuclear translocation of SREBP-1c.

### Phosphorylation of Ser372 on SREBP-1c Is Required for AST-Induced Repression of SREBP-1c in Transcription Activity in Insulin-Resistant HepG2 Cells

Ser372 on SREBP-1c has been confirmed as a major phosphorylation site for AMPK. To further investigate whether Ser372 phosphorylation is essential for AST-induced repression of SREBP-1c transcription activity in insulin-resistant HepG2 cells, we constructed lentivirus packaged SREBP-1c S372A wild-type plasmids and SREBP-1c S372A mutant plasmids, and used them to infect HepG2 cells for the purpose of creating stable cell lines.

**FIGURE 4:**
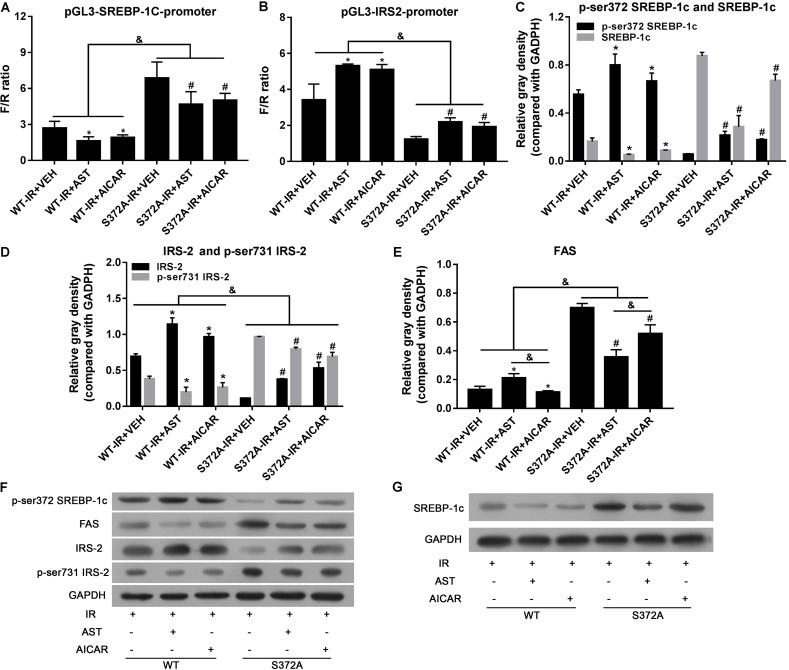
Ser372 phosphorylation was required for AST-induced repression of SREBP-1c transcription activity in insulin-resistant HepG2 cells. Mutation of SREBP-1c at Ser372 reversed the effect of AST on the transcription activity of SREBP-1c **(A)**, and decreased *IRS-2* transcription **(B)**; Mutation of SREBP-1c at Ser372 reversed the effect of AST on phosphorylation of SREBP-1c **(C)**, IRS-2 **(D)**, and expression of FAS **(E)**. **(F,G)** Representative bands from immunoblotting assays. AST, astragaloside; VEH, vehicle; IR, insulin resistance; WT, wild type. Data represent the mean ± SD as determined by one-way ANOVA, ^&^*p* < 0.05, ^∗^*p* < 0.05 vs. the WT-IR+VEH group; ^#^*p* < 0.05 vs. the S372A-IR+VEH group.

Dual luciferase reporter assays were performed to investigate the transcription activity of SREBP-1c, as well as transcription of its downstream target gene, *IRS-2*. As shown in **Figure 4A**, the transcription activity of SREBP-1c in the S372 group was significantly increased when compared with activity in the WT group (*p* < 0.05). Furthermore, when compared with the VEH group, AST or AICAR significantly decreased the transcription activity of SREBP-1c in both the WT group and S372 group (*p* < 0.05). *IRS-2* transcription in the S372 group was significantly lower than that in the WT group (**Figure 4B**, *p* < 0.05). Finally, when compared with the VEH groups, AST or AICAR significantly increased *IRS-2* transcription in both the WT group and S372 group (**Figure 4B**, *p* < 0.05). Western blotting assays for SREBP-1c, P-Ser372 SREBP-1c (**Figure 4C**), IRS-2, P-Ser731-IRS-2 (**Figure 4D**), and FAS (**Figure 4E**) revealed that mutation of SREBP-1c at Ser372 eliminated the effect of AST on expression of the FAS protein, which is expressed downstream of SREBP-1c (**Figure 4E**), and could partially blunt the effect of AST on IRS-2 phosphorylation and expression (**Figure 4D**). These changes are shown in **Figures 4F,G**.

Taken together, our results show that phosphorylation of Ser372 is crucial for the inactivation of SREBP-1c by AST, which activates AMPK by inducing phosphorylation of the AMPK α submit at Thr172.

### Phosphorylation of SREBP-1c at Ser372S Is Required for AST-Induced Improvements in Glucose Consumption and Suppression of Triglyceride Accumulation in Insulin-Resistant HepG2 Cells

**FIGURE 5:**
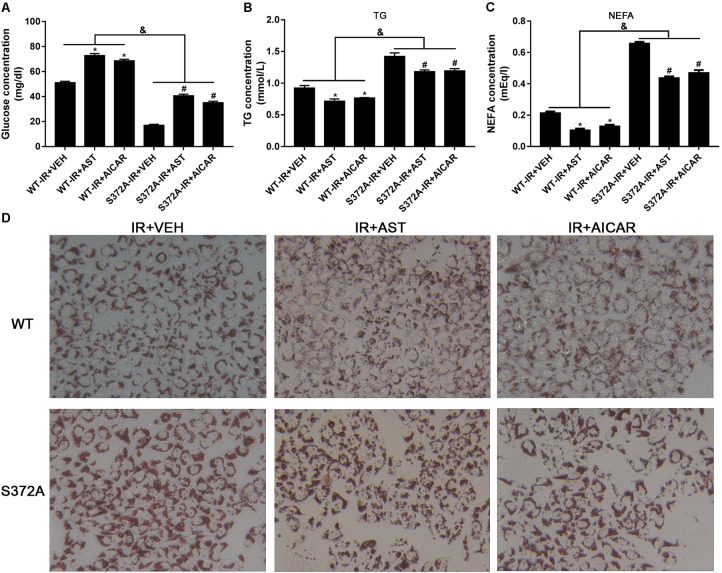
Ser372 phosphorylation was required for AST to reverse triglyceride accumulation and insulin resistance in HepG2 cells. Mutation of SREBP-1c at Ser372 reversed the effect of AST on glucose consumption **(A)** and TG **(B)** and NEFA production **(C)** in insulin-resistant HepG2 cells. **(D)** Representative gross morphology of a triglyceride accumulation as visualized by Oil Red O staining. Magnification: ×200^∗^. AST, astragaloside IV; VEH, vehicle; NEFA, non-esterified fatty acid; IR, insulin resistance; WT, wild type. Data represent the mean ± SD as determined by one-way ANOVA, ^&^*p* < 0.05, ^∗^*p* < 0.05 vs. the WT-IR+VEH group; ^#^*p* < 0.05 vs. the S372A-IR+VEH group.

Our glucose consumption assays showed that glucose concentrations in the WT group were considerably higher than those in cells transfected with mutant plasmids. Furthermore, AST or AICAR could significantly increase the glucose concentration in either group (**Figure 5A**).

Studies that evaluated NEFA and TG levels in the different groups revealed significantly increased levels of NEFAs and TGs in cells transfected with mutant plasmids when compared to cells transfected with wild-type plasmids. Furthermore, application of either AST or AICAR could reduce the TG (**Figure 5B**) and NEFA levels (**Figure 5C**).

Oil Red O staining was used to evaluate triglyceride accumulation in two stable HepG2 cells. As shown in **Figure 5D**, HepG2 cells in the WT-IR+VEH, WT-IR+AST, and WT-IR+AICAR groups displayed less triglyceride accumulation than HepG2 cells in the S372A-IR+VEH, S372A-IR+AST, and S372A-IR+AICAR groups. After treatment with AST or AICAR, significantly less dense triglyceride accumulations were found in cells transfected with either the wild-type plasmids or mutant plasmids, indicating that AST or AICAR could reduce triglyceride accumulation in HepG2 cells.

## Discussion

In this study, we investigated the effect of AST on regulating lipid accumulations in insulin-resistant HepG2 cells, and elucidated the underlying mechanism for the effect. We found that AST attenuated IR and lipid accumulation in HepG2 cells. As an AMPK activator, AST promoted *AMPK* gene expression and also activation of the AMPK protein by increasing phosphorylation of the protein. AST also inhibited the accumulation of nuclear SREBP-1c and transcription activity in insulin-resistant HepG2 cells by inducing phosphorylation of SREBP-1c at Ser372. These findings suggest AST as a promising therapeutic agent for treating hepatic steatosis.

In our study, we investigated the effect of AST on reducing IR in hepatocytes. The effect of AST on IR has been previously studied in other cell types, such as C2C12 myotubes (Zhu et al., 2016), endothelial cells (Xu et al., 2006; Jiang et al., 2008), and 3T3-L1 adipocytes (Zhao et al., 2015).

Sterol regulatory element binding protein-1c is the major transcription factor involved in lipogenesis in hepatocytes. To initiate the transcription of lipogenic genes *FAS* and *SCD1*, SREBP-1c translocates from the cytoplasm into the nucleus and then binds to the promoters of those genes. In our study, we found that HepG2 cells exposed to high glucose and insulin concentrations had increased levels of SREBP-1c in the nucleus and also displayed increased levels of downstream *FAS* and *SCD1* expression. Previous animal and clinical studies have reported increased levels of SREBP-1c in liver tissue (Li et al., 2011; Wu et al., 2016; Zhou et al., 2017). A study by Zhou et al. suggested that AST may attenuate FFA-induced lipid accumulation in hepatocytes by activating AMPK, as well as by inhibiting ER stress and SREBP-1c-mediated lipogenesis. The results of our study are consistent with those of Zhou. When compared with the study by Zhou et al., which investigated the dose-dependent effect of AMPK activation in a FFA-induced lipid accumulation model, our study focused on the biological effect of a low AST concentration on IR in hepatocytes.

Our study confirmed the role of SREBP-1c in IR in hepatocytes, and also investigated the effect of AST on the expression and phosphorylation of IRS-2 in insulin-resistant HepG2 cells. In hepatic cells, IRS-2 plays a crucial role in insulin signaling, as it regulates lipid metabolism (Taniguchi et al., 2005). SREBP-1c was found to suppress IRS-2 expression by binding to the promoter region of *IRS-2*, and thereby producing a type of IR (Ide et al., 2004) in which nuclear SREBP-1c decreases IRS-2 protein expression and increases autophosphorylation. Our study showed that AST could significantly reverse the expression and phosphorylation of IRS-2, and the underlying mechanism may be that AST inhibits SREBP-1c phosphorylation and nuclear translocation.

AMP-activated protein kinase consists of 3 submits: subunit α (α1, α2), submit β (β1, β2), and submit γ (γ1, γ2, γ3) (Mahlapuu et al., 2004). Phosphorylation of AMPKα1 at Thr172 creates the active conformation of AMPKα (Carling et al., 2012). Studies on the crystal structure of the AMPKα1 subunit have revealed that AMPKα1 contains an auto-inhibitory domain and a kinase domain. When high levels of AMPK become bound to the γ submit, the inhibitory domain of the α1 subunit is released from the kinase domain. This release allows for the phosphorylation of AMPKα at Thr172 by upstream kinases (Young, 2009).

In our study, we found that cells in the IR group that were exposed to high concentrations of high glucose and insulin had downregulated levels of AMPKα1 and AMPKα2 expression. Application of an AST and AMPK activator (AICAR) induced a significant increase in p-AMPKα1-Thr172 levels, suggesting that AST may serve as the AMPK activator. A further analysis revealed that application of AST induced decreases in TG and NEFA levels, as well as triglyceride accumulations in hepatocytes. A previous study showed that overexpression of AMPKa1 in the liver reduces lipogenic gene expression, liver triglyceride content, and hepatic steatosis in animals (Seo et al., 2009). AMPK phosphorylates downstream protein ACC1 at Ser79 (Su et al., 2012), and thereby reduces lipogenesis. In our study, ACC1 expression increased after cells were exposed to high levels of glucose and insulin, and such exposure decreased ACC1 phosphorylation at Ser79. In contrast, AST increased the levels of pSer79 ACC1 in HepG2 cells (**Supplementary Figure S1**). These findings confirmed the role of AST as an AMPK activator.

As a crucial molecule upstream of SREBP-1c, activation of AMPK will induce phosphorylation of SREBP-1c at Ser372, which in turn, will decrease the cleavage and nuclear translocation of SREBP-1c-Ser372 (Li et al., 2011; Hardie, 2015). Therefore, the AMPK/SREBP-1c pathway is likely involved in alleviation of hepatic lipid accumulation. Our study revealed that AST enhances phosphorylation of SREBP-1c at Ser372, and decreases the accumulation and nuclear translocation of p-SREBP1c-Ser372. Furthermore, we found that mutation of Ser372 completely inhibits the effects of AST on SREBP-1c expression, and phosphorylation of SREBP-1c at Ser372, and may affect the expression and activation of IRS-2 (**Supplementary Figure S2**). Our results provide new supportive evidence that AMPK activation by AST, and the subsequent inhibition of SREBP-1c-mediated lipogenesis, constitute a potential mechanism for the beneficial effect of AST on lipid accumulation and IR in hepatocytes.

In our study, we found that cell proliferation became inhibited at higher AST concentrations, but increased with incubation time (**Figure 1A**). These results suggest a mild anti-proliferative effect of AST on HepG2 cells. However, the effect of AST on cell proliferation is inconsistent across different studies, and depends on the cell stain, cell status, and culture conditions (Yuan et al., 2008; Zhang et al., 2011; Li et al., 2012; Ji et al., 2015). The concentrations of AST used in our study were relatively low when compared with those used in previous studies. We believe that a more significant inhibitory effect on cell proliferation would be induced by a high concentration of AST.

## Conclusion

This study proves that AST exerts a lipid-lowering effect in insulin-resistant hepatocytes by stimulating AMPK activation and decreasing phosphorylation of SREBP-1c at Ser372. These results indicate that AST can ameliorate hepatic steatosis, and suggest its potential as a drug for treating NASH.

## Author Contributions

CW designed the experiments and wrote the manuscript. CW, YL, and MH performed all the molecular biology experiments. WL revised the manuscript. All authors reviewed the manuscript.

## Conflict of Interest Statement

The authors declare that the research was conducted in the absence of any commercial or financial relationships that could be construed as a potential conflict of interest.

## Supplementary Material

FIGURE S1 Scanned picture of all western blotting bands.

FIGURE S2 Scanned picture of IRS and its phosphorylated bands detected by western blotting.

## Abbreviations

AMPK: AMP-activated protein kinase
AST: astragalosides A
C: compound C
CCK-8: Cell Counting Kit-8
DMEM: Dulbecco’s-modified Eagle’s medium
FAS: fatty acid synthase
HRP: horseradish peroxidase
IR: insulin resistance
IRS: insulin receptor substrate
NAFL: non-alcoholic fatty liver
NAFLD: non-alcoholic fatty liver disease
NASH: non-alcoholic steatohepatitis
NEFA: non-esterified fatty acid
nSREBP-1c: nuclear SREBP-1c
P-Ser372 SREBP-1c: Ser372 phosphorylated SREBP-1c
P-Ser731 IRS-2: Ser731 phosphorylated IRS-2
P-Ser79 ACC1: Ser79 phosphorylated ACC1
SCD1: stearoyl-coenzyme A desaturase 1
SREBP-1c: sterol regulatory element binding protein-1c
TG: triglyceride
VEH: Vehicle
WT: wild-type
