# Supplementary figures and images for: Astragaloside IV Inhibits Triglyceride Accumulation in Insulin-Resistant HepG2 Cells via AMPK-Induced SREBP-1c Phosphorylation
Source: Front Pharmacol. 2018 Apr 16;9:345. doi: 10.3389/fphar.2018.00345 (PMC5911465; doi:10.3389/fphar.2018.00345)

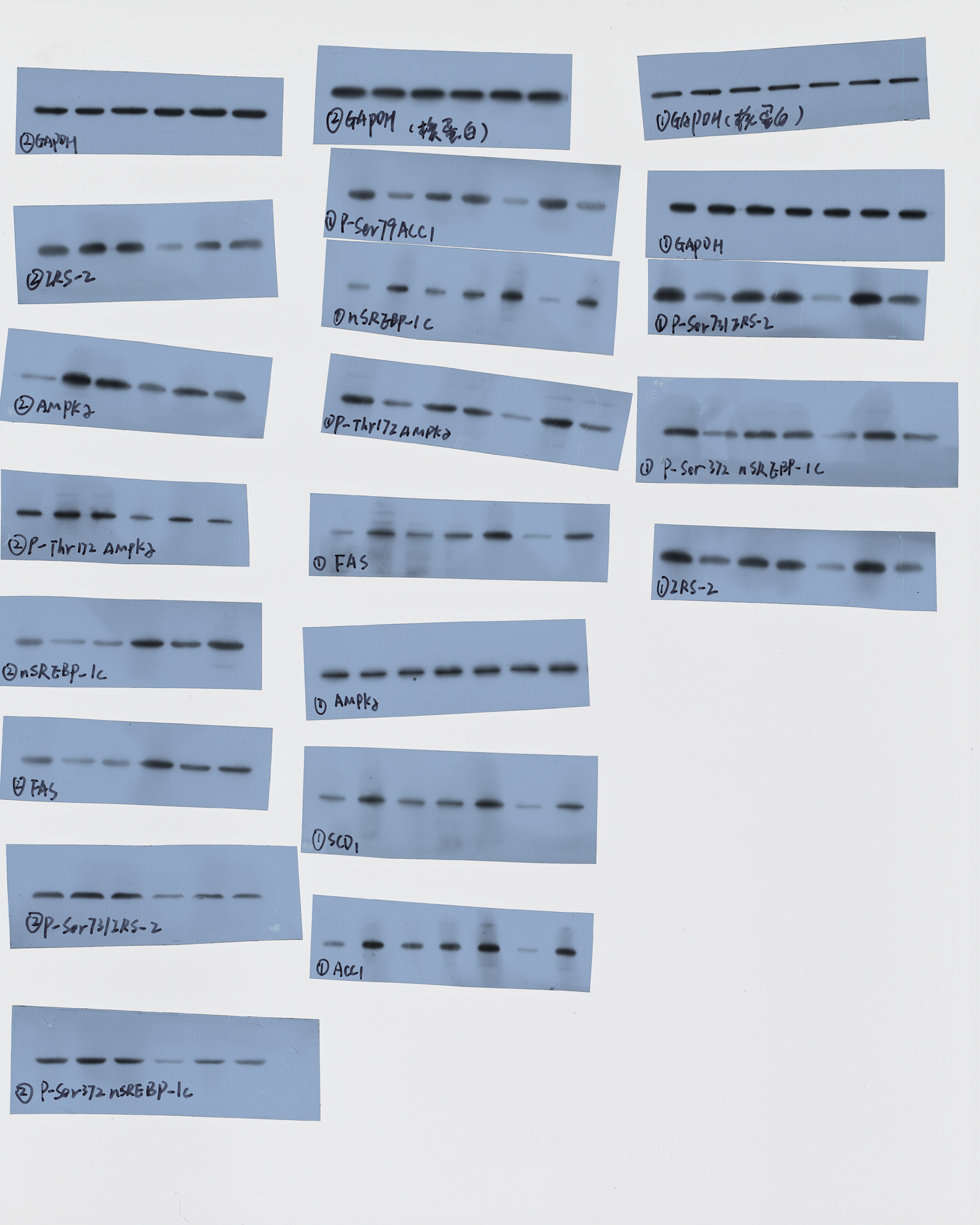

Supplement: FIGURE S1 — Scanned picture of all western blotting bands. [file Image_1.TIF]

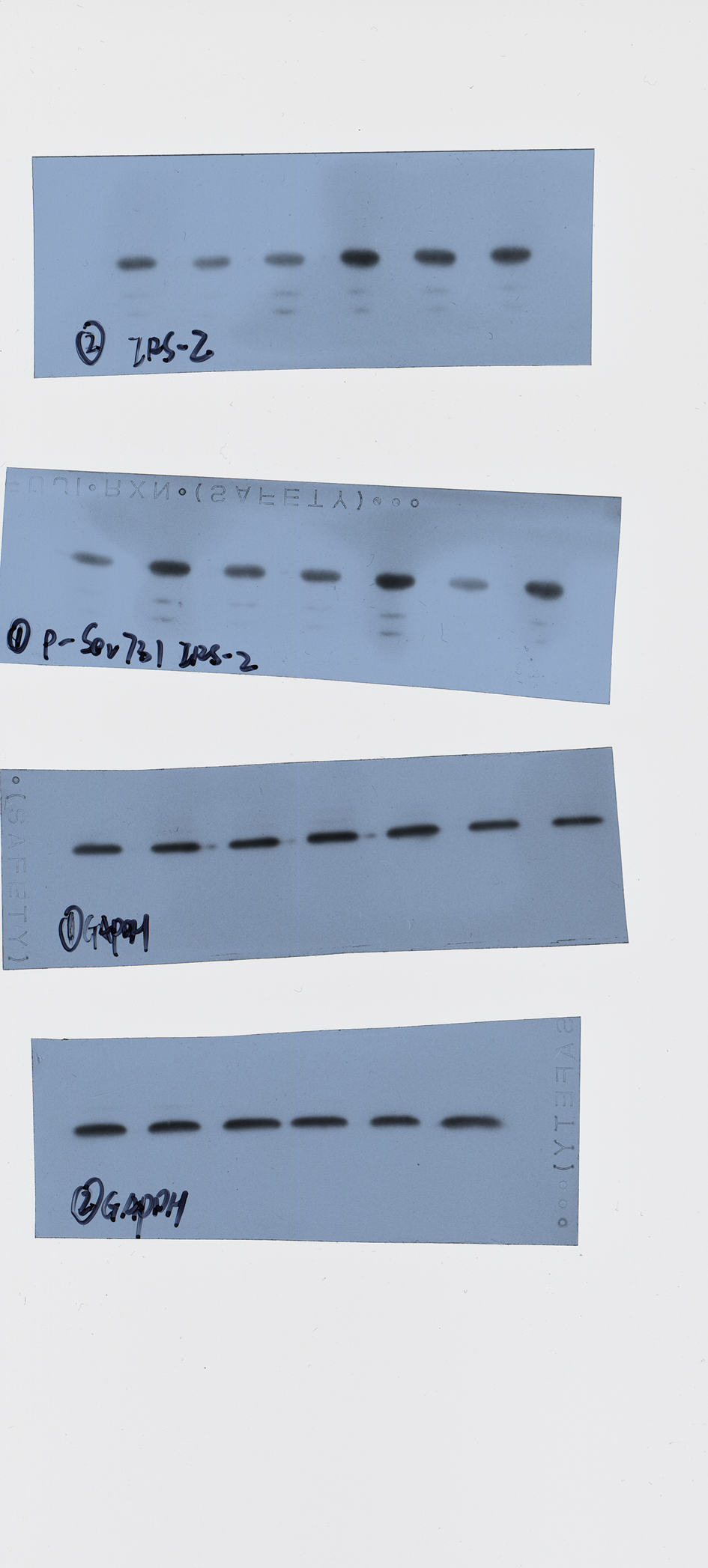

Supplement: FIGURE S2 — Scanned picture of IRS and its phosphorylated bands detected by western blotting. [file Image_2.TIF]
